# Characterization of mosquito host-biting networks of potential Rift Valley fever virus vectors in north-eastern KwaZulu-Natal province, South Africa

**DOI:** 10.1186/s13071-024-06416-0

**Published:** 2024-08-13

**Authors:** Takalani I. Makhanthisa, Milehna M. Guarido, Alan Kemp, Jacqueline Weyer, Melinda K. Rostal, William B. Karesh, Peter N. Thompson

**Affiliations:** 1 Department of Production Animal Studies, Faculty of Veterinary Science, University of Pretoria, Onderstepoort, South Africa; 2 Centre for Emerging Zoonotic and Parasitic Diseases, National Institute for Communicable Diseases of the National Health Laboratory Service, Sandringham, South Africa; 3 Department of Medical Virology, Faculty of Health Sciences, University of Pretoria, Pretoria, South Africa; 4 Clinical Microbiology and Infectious Diseases, School of Pathology, University of the Witwatersrand, Johannesburg, South Africa; 5 EcoHealth Alliance, New York, USA; 6 Centre for Veterinary Wildlife Research, Faculty of Veterinary Science, University of Pretoria, Onderstepoort, South Africa

**Keywords:** Rift Valley fever virus, Mosquito, Vertebrate blood meal sources, Network analysis, Arbovirus, South Africa

## Abstract

**Background:**

Rift Valley fever virus (RVFV) is a zoonotic mosquito-borne virus with serious implications for livestock health, human health, and the economy in Africa, and is suspected to be endemic in north-eastern KwaZulu-Natal (KZN), South Africa. The vectors of RVFV in this area are poorly known, although several species, such as *Aedes* (*Neomelaniconion*) *mcintoshi*, *Aedes* (*Neomelaniconion*) *circumluteolus*, *Aedes* (*Aedimorphus*) *durbanensis*, and *Culex* (*Lasioconops*) *poicilipes* may be involved. The aim of the study was to determine the vertebrate blood meal sources of potential RVFV mosquito vectors in north-eastern KZN and to characterize the host-biting network.

**Methods:**

Blood-fed mosquitoes were collected monthly from November 2019 to February 2023 using a backpack aspirator, CO_2_-baited Centers for Disease Control and Prevention (CDC) miniature light traps and tent traps, in the vicinity of water bodies and livestock farming households. The mosquitoes were morphologically identified. DNA was extracted from individual mosquitoes and used as templates to amplify the vertebrate cytochrome *c* oxidase I (*COI*) and cytochrome *b* (*cytb*) genes using conventional polymerase chain reaction (PCR). Amplicons were sequenced and queried in GenBank and the Barcode of Life Data systems to identify the vertebrate blood meal sources and confirm mosquito identifications. All mosquitoes were screened for RVFV using real time reverse transcription (RT)-PCR.

**Results:**

We identified the mammalian (88.8%) and avian (11.3%) blood meal sources from 409 blood-fed mosquitoes. *Aedes circumluteolus* (*n* = 128) made up the largest proportion of collected mosquitoes. Cattle (*n* = 195) and nyala (*n* = 61) were the most frequent domestic and wild hosts, respectively. Bipartite network analysis showed that the rural network consisted of more host-biting interactions than the reserve network. All mosquitoes tested negative for RVFV.

**Conclusions:**

Several mosquito species, including *Ae*. *circumluteolus*, and vertebrate host species, including cattle and nyala, could play a central role in RVFV transmission. Future research in this region should focus on these species to better understand RVFV amplification.

**Graphical Abstract:**

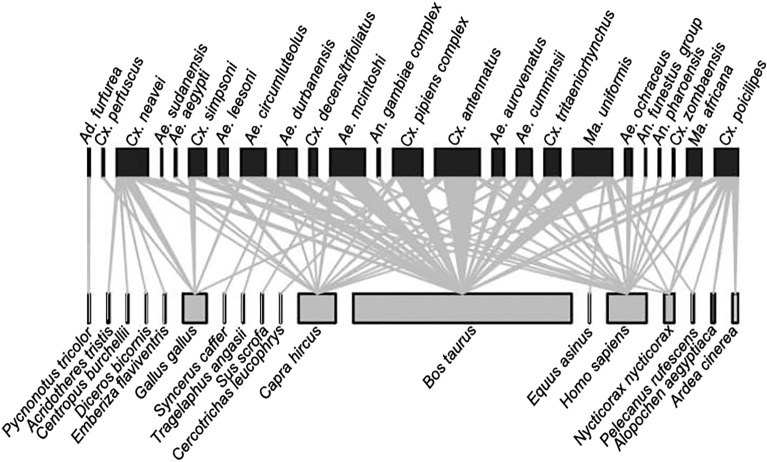

**Supplementary Information:**

The online version contains supplementary material available at 10.1186/s13071-024-06416-0.

## Background

Rift Valley fever virus (RVFV) (genus *Phlebovirus*, family Phenuiviridae [1]) is a single stranded RNA zoonotic mosquito-borne virus that causes periodic outbreaks with significant veterinary health, public health, and economic consequences [2, 3]. The maintenance and survival of RVFV during interepidemic periods is poorly understood and is thought to be via a combination of low level circulation between mosquito vectors and vertebrate hosts, and vertical (transovarial) transmission, where the virus is passed from infected adult mosquitoes to progeny via eggs [4]. Floodwater-breeding *Aedes* spp. mosquitoes, mainly of the subgenera *Neomelaniconion* and *Ochlerotatus* are the suspected primary vectors that potentially transmit the virus both transovarially and horizontally [5]. These mosquitoes lay drought-resistant eggs in low-lying flooded grasslands, edges of water surfaces or pans (dambos) that are prone to flooding, which hatch after temporary desiccation following subsequent flooding [6]. Transovarial transmission has been proposed in *Ae*. (*Neomelaniconion*) *mcintoshi* [7] and was suggested to likely occur in other floodwater-breeding *Aedes* species, such as *Aedes* (*Aedimorphus*) *vexans* [6]. Mosquitoes from other genera, such as *Culex*, *Anopheles*, and *Mansonia*, are classified as secondary vectors that amplify RVFV and transmit it horizontally when environmental conditions conducive for high vector concentrations persist [7].

In South Africa, the first recorded Rift Valley fever (RVF) outbreak occurred in 1950–1951 and resulted in an estimated 500,000 abortions and 100,000 deaths in sheep [8]. Since then, other outbreaks affecting both livestock and humans have been reported in various parts of the country. Larger outbreaks have been reported in the central interior of the country in the Free State, Northern Cape, and Eastern Cape provinces, while smaller outbreaks were noted in Mpumalanga, Gauteng, and KwaZulu-Natal (KZN) [9]. The most recent widespread outbreaks in South Africa were reported in 2010–2011, in the central interior of South Africa [9]. However, in Maputaland in far north-eastern KZN, substantial endemic RVFV circulation has recently been demonstrated, with a seroprevalence of 34% and 32% in cattle and goats, respectively [10]. *Aedes mcintoshi* and *Cx*. (*Culex*) *theileri* are considered to be important RVFV vectors on the inland plateau while *Ae*. (*Neomelaniconion*) *circumluteolus* and *Cx*. (*Lasioconops*) *zombaensis* have been reported to be significant vectors in the coastal lowlands of KZN [5]. Previous work in far north-eastern KZN [11], suggests that RVFV vectors may include *Ae*. *mcintoshi*, *Ae*. *circumluteolus*, *Ae*. (*Aedimorphus*) *durbanensis*, *Ae*. (*Aedimorphus*) *ochraceus*, *Cx*. (*Culex*) *tritaeniorhynchus*, and *Cx*. *poicilipes*. Potential vectors of a virus include mosquito species that are abundant in an area where it is present, are susceptible to it and have demonstrated the ability to successfully transmit it by bite [12].

It is unclear which hosts provide blood meals to RVFV mosquito vectors. Vertebrate blood meal sources can vary by mosquito species, geographic location, availability of vertebrate hosts, and environmental conditions [13]. Previously, analysis of mosquito blood meal sources in Kenya indicated that *Ae*. *mcintoshi* and *Ae.*. *circumluteolus* mainly fed on cattle rather than on humans and other available hosts [14]. Another study from Kenya indicated that the vertebrate blood meal sources of primary and secondary mosquito vectors for Rift Valley fever virus (RVFV) encompassed a range of animals, such as cattle, goats, sheep, humans, camels, and donkeys [15]. A study conducted in the coastal region of KZN in South Africa showed that cattle were the most frequent vertebrate hosts of *Cx*. *zombaensis* [16]. Another early study conducted on mosquitoes from Ndumo Game Reserve (NGR) in northern KZN, reported that *Ae*. *circumluteolus* mainly fed on larger mammals while *Cx*. (*Culex*) *neavei* fed mostly on birds, rodents, and hares [17]. Moreover, in NGR, a study used human, monkey, and fowl baits to show that humans were the preferential baits for *Ae*. *circumluteolus* and *Cx*. (*Culex*) *antennatus* and *Ae*. *durbanensis* preferred human bait while *Cx*. *neavei* favored fowl bait [18]. A recent study in northern KZN reported that *Ae*. *circumluteolus* fed on cattle and *Ae*. *durbanensis* on cattle, goats, and sheep [19]. Knowledge of blood meal host sources is also important for potential RVFV horizontal vectors from other genera, such as *Cx*. and *Mansonia*, as they play a significant role in amplifying the virus [20]. Cattle, goats, and sheep have been found to be significant amplifiers of RVFV; however, a substantial gap still exists in understanding the diversity of the hosts of RVFV vectors [20].

Host specialization for blood-feeding mosquitoes can vary from vector to vector and geographically. Network ecology provides a useful tool to study such contributions within host-biting communities and, thus, provides insights on disease transmission dynamics [21]. Bipartite networks have been used recently to study the interconnections of host-biting communities and they provide a visual representation of interactions between species (nodes) connected by edges (feeding interactions) [21]. This network tool can be used to identify potentially significant vectors and to describe the role of vertebrate hosts that are part of the transmission cycle [22]. An added benefit of the bipartite network analysis is that it does not require data regarding the availability of hosts from the study areas as an input [22].

Immunological techniques, such as enzyme-linked immunosorbent assays (ELISA), were previously used to determine blood meal source species [23]. Although these immunological methods were valuable, the partial degradation of blood products, such as proteins, peptides, and antibodies, over time was a challenge [24]. A more recent polymerase chain reaction (PCR) method used to identify the host involves amplification of cytochrome *c* oxidase subunit I (*COI*) or cytochrome *b* (*cytb*) genes from mitochondrial DNA extracted from the abdomen of blood-fed mosquitoes [25]. These genes are reliable and commonly used targets for identifying arthropod blood meals because they exist in hundreds to thousands of copies per cell and contain independent genomes [26, 27]. PCR assay methods and sequencing of the amplicons to identify the vertebrate blood meal sources have improved greatly over the years [28]. However, identification is limited to species for which nucleotide sequences are available on the GenBank database (https://www.ncbi.nlm.nih.gov/genbank/).

The aim of this study was to determine the vertebrate blood meal sources of potential RVFV mosquito vectors in north-eastern KZN and to characterize the host-biting network. The specific objectives were: (i) to identify the vertebrate blood meal sources of mosquitoes collected from a wildlife reserve and an adjacent rural livestock farming area, (ii) to evaluate and compare the patterns of vertebrate blood host selection by mosquitoes from the reserve and rural settings using bipartite network analysis, and (iii) to detect RVFV in blood-fed mosquitoes using real time reverse-transcription PCR (RT-PCR).

## Methods

### Study area

The study was conducted on the Maputaland Coastal Plain, in far north-eastern KZN, South Africa, which extends into southern Mozambique. It is a humid area characterized by dry, warm winters with temperatures ranging from 16 to 26 °C and hot, wet summers [29]. The area has an average annual temperature range of 23–40 °C, and mean annual rainfall of 600–800 mm [29] with most precipitation falling between October and March [30]. The northwards flowing Phongolo River that forms a floodplain along the eastern foot of the Lebombo mountains [31] is an important region of the study area. The Phongolo, Ingwavuma, and Usuthu Rivers flood seasonally based on the amount of rainfall, covering about 13,000 ha of the floodplain and filling many pans, some of which retain water during dry season [32]. However, the construction of the Pongolapoort Dam in 1973 altered this cycle, limiting flooding events to heavy rainfall or the periodic opening of the dam’s sluices [33]. As a result, during very dry years, permanent water may only be found in the Phongolo and Usuthu Rivers and a few large pans.

Sampling was conducted along pans situated 8 km apart at Namaneni (−26.986765, 32.275357) and Mpala (Qotho Pan, −26.941284, 32.216295). The study began in November 2019, during a drought period when both pans were dry, with small temporary areas of flooding during summer, and continued after the 2021 flooding that left both pans with large amounts of water year-round. These pans are situated in a communal farming area with a high density of humans, cattle, and goats. In addition, mosquitoes were aspirated adjacent to a livestock-owning household located about 6 km from NGR and mid-way between the two pans. Blood-fed mosquitoes were also collected from Ndumo Game Reserve (NGR; −26.880935, 32.251996), a 10,117 ha wildlife reserve bordering Mozambique to the north [34]. The reserve is home to many animals including crocodiles, hippos, bushpigs, warthogs, and various antelopes [35].

### Blood-fed mosquito sampling

Blood-fed mosquitoes were collected monthly (January 2022–February 2023) using a Centers for Disease Control and Prevention (CDC) backpack aspirator (John W. Hock Company, USA) operated by a motor, aspirating mosquitoes into a 100 mm diameter cup. Additional blood-fed mosquitoes were obtained from among mosquitoes collected monthly (November 2019–November 2022) using carbon dioxide (CO_2_)-baited net traps and modified (without light source) CO_2_-baited CDC miniature light traps (BioQuip, USA). The collected mosquitoes were killed with dry ice (solid CO_2_) and stored in 15 ml plastic tubes, which were then stored in polystyrene boxes containing dry ice and later transferred to a −20 °C portable freezer. Blood-fed mosquitoes were distinguished from non-blood-fed mosquitoes based on their abdomen, which becomes engorged and reddish to black for several hours after feeding, separated and stored individually in 2 ml cryotubes. The mosquitoes were then transported to the University of Pretoria, Faculty of Veterinary Science, Department of Veterinary Tropical Diseases (DVTD) Research and Training Laboratory, where they were stored at −20 °C until identification. The blood-fed mosquitoes were morphologically identified under a stereo-microscope (zoom magnification = 15–40 ×) using available taxonomic keys [36, 37] and stored individually in 2 ml cryotubes at −80 °C until further processing.

### Molecular identification of vertebrate blood meal hosts

Blood-fed mosquitoes stored individually in 2 ml cryotubes were homogenized in 100 µl Eagle’s minimal essential medium (EMEM) culture medium (BioWhittaker^®^, Lonza, USA) through high-speed shaking for 4 min at 30 Hz in a TissueLyser||(QIAGEN, USA) and centrifuged for 2 min at 4722 rpm. Genomic DNA was extracted from the homogenized mosquitoes using prepGEM Universal kits (MicroGEM, UK) following the manufacturer’s instructions. Extracted DNA samples were used as templates in a conventional PCR to amplify the *COI* gene using the VF1d_t1 and VR1d_dt primer sequences (Table 1) targeting a 750 bp region [38]. The PCR cycling conditions consisted of initial denaturation at 98 °C for 1 min, 40 cycles of denaturation, annealing and extension at 98 °C for 10 s, 57 °C for 30 s, and 72 °C for 30 s, respectively, and a final elongation step at 72 °C for 7 min [15]. A 23 µl reaction volume consisting of 10 µl Phusion flash high-fidelity PCR master mix (ThermoFisher Scientific, Lithuania), 0.5 µl of each primer (20 µM), 5.5 µl DNA template, and 6.5 µl ddH_2_O was used.

**Table 1:** Primers and sequences used for *COI* and *cytb* conventional PCR, and real time RT-PCR methods

Primer	Sequence (direction, target gene)
VF1d_t1	5′-TGTAAAACGACGGCCAGTTCTCAACCAACCACAARGAYATYGG-3′ (forward, vertebrate *COI*)
VR1d_dt	5′-CAGGAAACAGCTATGACTAGACTTCTGGGTGGCCRAARAAYCA-3′ (reverse, vertebrate *COI*)
L14841	5′-CCATCCAACATCTCAGCATGATG AAA-3′ (forward, vertebrate *cytb*)
H151494	5′-GCCCCTCAGAATGA TATTTGTCCTCA-3′ (reverse, vertebrate, *cytb*)
LCO1490	5′-GGTCAACAAATCATAAAGATATTGG-3′ (forward, mosquitoes *COI*)
HCO2198	5′-TAAACTTCAGGGTGACCAAAAAATCA-3′ (reverse, mosquitoes *COI*)
Sense	5′-AAAGGAACAATGGACTCTGGTCA-3′ (forward, mosquitoes G2)
Antisense	5′-CACTTCTTACTACCATGTCCTCCAAT-3′ (reverse, mosquitoes G2)
Probe	5′-AAAGCTTTGATATCTCTCAGTGCCCCAA-3′ (5′-nuclease probe)

Cytochrome b (*cytb*) conventional PCR was only conducted if the *COI* primer pair did not amplify the blood meal DNA. The L14841 and H151494 primers (Table 1) used for this PCR targeted a 358 bp region of the *cytb* gene [15, 39]. The PCR conditions included a cycle of initial denaturation for 1 min at 98 °C, 40 cycles of denaturation, annealing and extension at 98 °C for 30 s, 61 °C for 20 s, and 72 °C for 30 s, respectively, and a final extension step of 72 °C for 7 min. The PCR reaction volumes and reagents were the same as those used for the *COI* PCR described above, using *cytb* PCR primers at similar concentrations.

### Molecular identification of mosquitoes

An additional conventional PCR with primers (Table 1) targeting a 710 bp fragment of the *COI* [40] of insects was performed to confirm morphological identifications of damaged mosquitoes. A total reaction volume of 23 µl consisted of 10 µl Phusion flash high-fidelity PCR master mix (ThermoFisher Scientific, Lithuania, Europe), 0.5 µl of both the LCO1490 and HCO2198 primers (20 µM), 5.5 µl DNA template and 6.5 µl ddH_2_O. The PCR mixture was subjected to the following PCR thermal cycling conditions: 98 °C for 10 s, 30 cycles of 98 °C for 1 s, 55 °C for 5 s, 72 °C for 15 s, and 70 °C for 1 min [40].

### Agarose gel electrophoresis and sequencing

The PCR products were resolved in a 1.5% agarose gel in Tris–borate ethylenediaminetetraacetic acid (EDTA) buffer stained with ethidium bromide. Agarose gel electrophoresis was conducted at 100 V for 75 min and the gels were visualized on a ultraviolet (UV) transilluminator and images were taken using Gel Documentation system (Bio-Rad, USA). The unpurified amplicons were sent to Inqaba Biotec (South Africa) for Sanger sequencing. The sequences were edited in Chromas gene editing software (version 2.6.6) and the edited sequences were queried against the GenBank DNA sequence database (https://www.ncbi.nlm.nih.gov/genbank/) using the Basic Local Alignment Search Tool (BLAST) and/or Barcode of Life Data (BOLD) systems (https://www.boldsystems.org/) to identify the blood meal hosts and mosquito species.

### Rift Valley fever virus detection using RT-PCR

The mosquitoes were screened for RVFV using a real time RT-PCR method that combines superscript reverse transcriptase with Taq-polymerase (Roche, USA) and Taqman^®^ probes), adapted from Drosten et al., as detailed below. Mosquitoes individually homogenized for DNA extraction (see above) were pooled (*n* ≤ 5) in 2 ml cryotubes by species, site, and sampling date. Nucleic acids were extracted from the pooled mosquitoes using the MagMAX™ total nucleic acid isolation kit (Applied Biosystems, USA). The lysis/binding solution was prepared in the sample preparation deep 96 well plate by combining the lysis/binding solution concentrate (232 µl per well) and carrier RNA (1 µg, 3 µl per well). The tubes containing the pooled homogenized mosquitoes were centrifuged for 2 min at 4722 rpm and 175 µl of clear supernatants were added to their respective wells in the deep 96 well plate containing the lysis/binding solution. Nucleic acids were then extracted from the samples using the kit and the KingFisher™ Flex instrument (ThermoFisher Scientific, USA) according to manufacturer’s instructions, and stored at −20 °C for RT-PCR use.

Real time RT-PCR was carried out in a LightCycler^®^ 96 instrument (Roche, USA) targeting the G2 gene with an amplicon length of 92 bp. A total RT-PCR reaction volume of 20 µl consisted of 2 µl (10 µM) of both the sense primer (Table 1) and the antisense primer (Table 1), 0.4 µl (10 µM) 5′-nuclease probe (Table 1), 0.4 µl taq polymerase (Roche, USA), 1.6 µl MgCl_2_, 4 µl reaction mix, 4.6 µl nuclease free water, and 5 µl of RNA template. The RT-PCR conditions consisted of reverse transcription for 30 min at 45 °C, HotStartTaq activation for 5 min at 95 °C, two steps of amplification for 5 s at 95 °C (45 cycles) and 35 s at 57 °C, and a final step of cooling for 30 s at 30 °C [41].

### Statistical analysis

#### Blood-meal identification

The vertebrate blood meal host sequences were edited in Chromas gene editing software (version 2.6.6) and the edited sequences were queried against DNA sequence from the BOLD system for *COI* sequences and the GenBank database using Basic Local Alignment Search Tool (BLAST) for *cytb* sequences. A *COI* identification search with the “Species Level Barcode Records” option was used on the BOLD system. For GenBank, a nucleotide BLAST search was performed using MegaBlast search option, which is optimized for highly similar sequences. A 96–100% identity was used for positive identification [15, 42] and a species was only considered accurately identified if the taxonomic name had the top match statistic. Tables were generated using Microsoft Excel (version 2021).

#### Bipartite network analysis

A bipartite network analysis was conducted to evaluate host-biting interactions for blood-fed mosquitoes collected from the wildlife reserve and rural sites. Indices were calculated to characterize the host-biting interactions from the reserve and rural sites. The connectivity calculates the average number of interactions per species in a network. The connectance is defined as the realized proportion of possible links and it is calculated as the fraction of links present in the network out of all possible links [21]. Other parameters that were evaluated included the species degree that measures the number of times each node has a connection, and the edge density, that measures the number of possible edges in a network [43]. Species strength and discrimination (*d*') indices were calculated to characterize some ecological traits for each mosquito species within the networks [22]. The species strength quantifies the relevance of a species across all its partners within a network and it is calculated as the sum of dependencies of a species ranging between 0 (minimum relevance) and the number of species in other group (maximum relevance) [44]. The *d*' index calculates how strongly a species deviates from a random sampling of interacting partners available and produces values between 0 (no specialization) and 1 (perfect specialist), indicating whether a mosquito species feeds on a common or rare host [45]. The *d*' value will tend to be lower for a mosquito that fed on a common vertebrate host and tend to be higher for a mosquito that fed on a rare host in the network. Bipartite network analysis was conducted using bipartite and igraph packages in R version 4.2.3.

#### Phylogenetic analysis

To further confirm the mosquito identifications of the damaged mosquitoes, phylogenetic analysis was conducted. The generated *COI* 710 bp sequences were used to generate a likelihood tree using the Molecular Evolutionary Genetics analysis (MEGA) system version 11.0.13 [46]. The homologous *COI* sequences with 96–100% similarity to the query sequences were downloaded from National Center for Biotechnology Information (NCBI) in FASTA format and loaded to the MEGA system together with the query sequences, then aligned using the MUSCLE option with the default settings. Different methods were explored to generate the phylogenetic tree and the maximum likelihood statistical method with the Tamura-Nei model was identified as the best fit. The branch probabilities of the ML phylogenetic tree were assessed using the bootstrap test of phylogeny method with 1000 replicates. One sand fly (*Lutzomyia longipalpis*) DNA sequence from the GenBank was included as an outgroup. Initial trees for the heuristic search were obtained automatically by applying neighbor-join and BioNJ algorithms to a matrix of pairwise distances estimated using the Tamura-Nei model, and then selecting the topology with superior log likelihood value. The tree was drawn to scale, with branch lengths measured in the number of substitutions per site. This analysis involved 63 nucleotide sequences. All codon positions, first, second, and third were included together with the noncoding sites. There were a total of 1485 positions in the final dataset. Evolutionary analyses were conducted in MEGA11 [46].

## Results

A total of 561 blood-fed mosquitoes were collected from rural areas (*n* = 445, 79.3%) and Ndumo Game Reserve (*n* = 116, 20.7%). Mosquitoes collected from the rural areas were more diverse and consisted of all 28 mosquito species that were collected in the study, while only 6 species were collected from the reserve (Appendix 1). The blood-fed mosquitoes belonged to seven genera: *Aedes* (*n* = 252), *Culex* (*n* = 230), *Mansonia* (*n* = 65), *Anopheles* (*n* = 11), *Aedeomyia* (*n* = 1), *Coquillettidia* (*n* = 1), and *Mimomyia* (*n* = 1) (Appendix 1). Most (86.1%) of the blood-fed mosquitoes were potential RVFV vectors including *Ae*. *circumluteolus* (*n* = 128, 22.8%), *Cx*. *antennatus* (*n* = 57, 10.2%), *Ae*. *mcintoshi* (*n* = 54, 9.6%) and *Cx*. *neavei* (*n* = 48, 8.6%). The other potential RVFV vectors were *Ae*. *durbanensis*, *Ae*. *ochraceus*, *Ae*. (*Aedimorphus*) *cumminsii*, *Ae*. (*Stegomyia*) *aegypti*, *Mansonia* (*Mansonioides*) *uniformis*, *Mansonia* (*Mansonioides*) *africana*, *Cx*. *poicilipes*, *Cx*. *tritaeniorhynchus*, *Cx*. *zombaensis*, and those from the *Cx*. *pipiens* complex. The majority (*n* = 91) of the *Ae*. *circumluteolus* were collected from the reserve, while most *Ae*. *mcintoshi* (*n* = 52) and all *Cx*. *antennatus* were collected from the rural areas (Appendix 1). The mosquitoes from the rural areas were collected from pans at Namaneni (*n* = 201, 45.2%) and Mpala (*n* = 44, 9.9%) and a household (*n* = 200, 44.9%).

A total of 409 (72.9%) blood-fed mosquitoes from reserve (*n* = 95) and rural areas (*n* = 314) belonging to five genera and 24 species were successfully analyzed to identify mammalian and avian blood meal sources (Table 2). Overall, mammalian hosts (*n* = 363, 88.8%) were more frequently identified than avian hosts (*n* = 46, 11.2%). Avian hosts were identified from the rural areas at all three sampling sites (Namaneni = 27, Mpala = 6, and household = 13). Nyala (*Tragelaphus angasii*) was the most frequent host from the reserve (*n* = 61) and cattle (*Bos taurus*) was the most common vertebrate blood meal source from the rural area (*n* = 195). Only three types of hosts, namely chicken (*Gallus gallus*), cattle, and human (*Homo sapiens*), were identified as vertebrate blood meal sources of *Ae*. *circumluteolus* in the rural area (Table 2). The vertebrate blood meal sources of *Ae*. *circumluteolus* from the reserve were more diverse, including nyala, buffalo (*Syncerus caffer*), hippopotamus (*Hippopotamus amphibius*), impala (*Aepyceros melampus*), giraffe (*Giraffa camelopardalis*), and human. All blood-fed *Ae*. *durbanensis* mosquitoes (*n* = 16) were collected from the household and their identified vertebrate blood meal sources were cattle (*n* = 12) and one each of human, goat (*Capra hircus*), buffalo, and nyala. The vertebrate blood meal sources of *Ae*. *mcintoshi* from the rural setting included cattle (*n* = 25), goat (*n* = *4*), human (*n* = 1), and White-browed Scrub Robin (*Cercotrichas leucophrys*, *n* = 1). *Aedes mcintoshi* from the reserve fed on buffalo, common duiker (*Sylvicapra grimmia*), impala, nyala, and suni (*Neotragus moschatus*). Other mammalian blood meal sources that were identified for potential RVFV vectors from the rural area were pig (*Sus scrofa*) and black rhinoceros (*Diceros bicornis*) (Table 2).

**Table 2:**
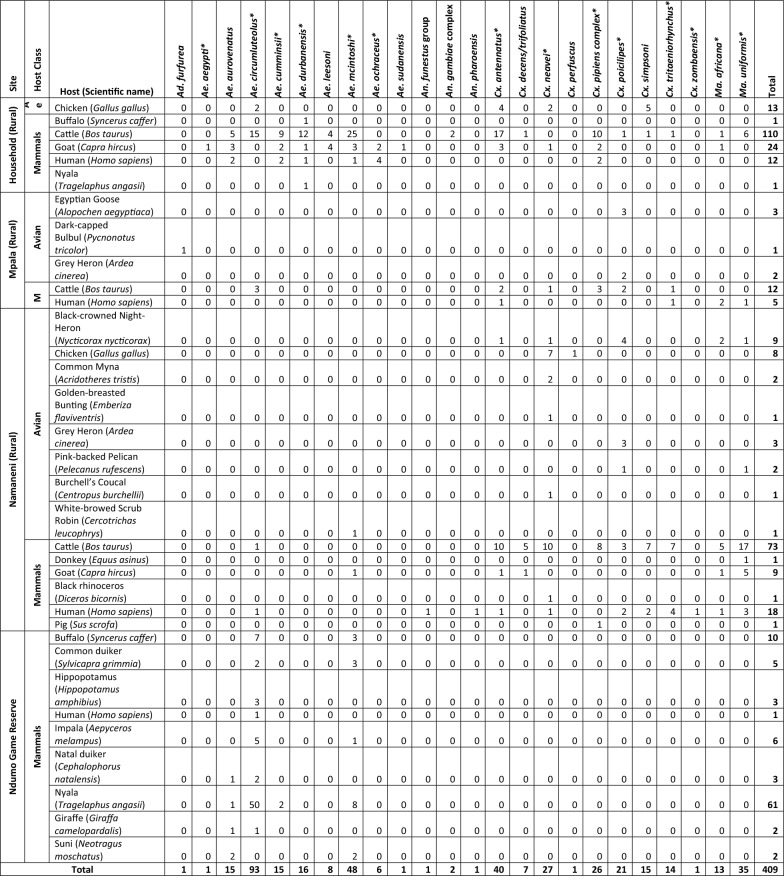
Numbers of mosquitoes with avian (A) and mammalian (M) blood meals from the wildlife reserve and rural areas (Namaneni, Mpala, household)

Potential RVFV mosquito vectors in the study area are indicated by *

Ten avian species were identified as blood meal sources, with the most frequent being chickens (*n* = 21) and Black-crowned Night Heron (*Nycticorax nycticorax*, *n* = 9). The potential RVFV vectors in the study areas that fed on avian hosts included *Ae*. *circumluteolus*, *Ae*. *mcintoshi*, *Cx*. *antennatus*, *Cx*. *neavei*, and *Cx*. *poicilipes*. *Cx*. *neavei* had the highest diversity of avian hosts which included chickens, Black-crowned Night Heron, Common Myna (*Acridotheres tristis*), Golden-breasted Bunting (*Emberiza flaviventris*), and Burchell’s Coucal (*Centropus burchellii*) (Table 2). Of the 152 (27.1%) blood meals that were not identified, 126 (22.5%) failed to amplify while 26 (4.6%) were amplified and sequenced but yielded a similarity percentage lower than the predetermined cutoff value of 96%.

All blood-fed mosquitoes tested negative for RVFV using real time RT-PCR.

### Bipartite network analysis

**Fig. 1:**
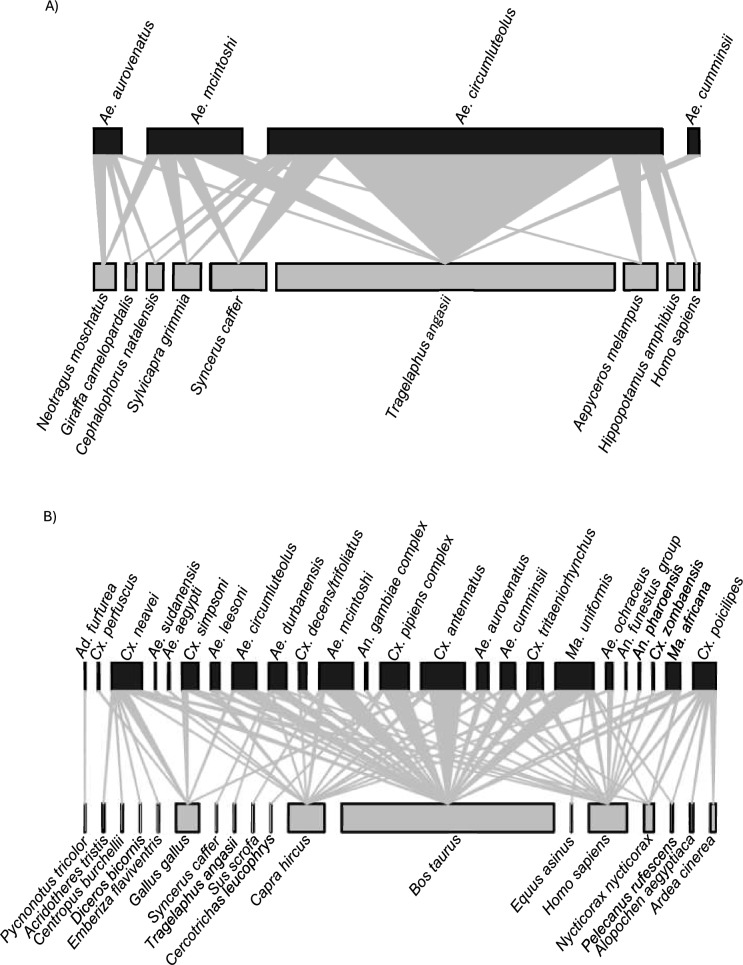
Bipartite network of blood-fed mosquitoes (upper) and their vertebrate blood meal sources (lower) collected from the wildlife reserve (**A**) and rural (**B**) areas in north-eastern KZN. The sizes of the upper and lower rectangles are proportional to the numbers of blood-fed mosquitoes and identified vertebrate hosts, respectively. The triangles connecting the mosquito and vertebrate host nodes represent interactions, with their width proportional to the frequency of the interactions

The rural network consisted of a higher number of interactions and more nodes both in the mosquito and vertebrate levels than the wildlife reserve network (Fig. 1 and Additional file 1: Fig. S1). The rural network was composed of 42 species (24 mosquitoes and 18 hosts) and the reserve network consisted of 13 species (4 mosquitoes and 9 hosts). The rural network included 314 interactions, with connectivity of 7.5, connectance of 0.2, and edge density of 0.4. The reserve network consisted of 95 interactions, with connectivity of 7.3, connectance of 0.6, and edge density of 1.2. The species with the highest number of connections in the rural network were cattle and *Cx*. *antennatus* with 195 and 40 connections, respectively. *Aedes circumluteolus* and nyala had the highest number of connections in the reserve network with 71 and 61 connections, respectively (Fig. 1).

The mosquitoes with the highest species strength in the rural network were *Cx*. *neavei* and *Cx*. *poicilipes*, with species strength of 4.65 and 3.03, respectively (Table 3). *Aedes durbanensis* and *Ae*. *mcintoshi* were the strongest *Aedes* mosquitoes in the rural network, with species strength of 2.1 and 1.3, respectively (Table 3). The strongest mosquito species in the reserve network was *Ae*. *circumluteolus*, with species strength of 5.9 (Table 3). Excluding *Aedeomyia* (*Lepiothauma*) *furfurea* (*n* = 1), mosquitoes from both the reserve and rural sites did not show high levels of specialization as their *d*' values were low, with the highest being 0.44 for both *Ae*. *aurovenatus* from the reserve and *Cx*. *poicilipes* from the rural network (Table 3). Only *Ae*. *circumluteolus* fed on the same host (human) in both the reserve and rural settings (Fig. 1). There were no mosquitoes that fed exclusively on avian hosts besides *Ad*. *furfurea* and *Cx*. (*Culex*) *perfuscus*, each only with one host-biting interaction with Dark-capped Bulbul (*Pycnonotus tricolor*) and chicken, respectively (Fig. 1).

**Table 3:** Species-level index values for species strength and discrimination (*d*') for blood-fed mosquitoes from rural and reserve networks

Species	Rural			Reserve		
	*n*	Strength	*d*'	*n*	Strength	*d*'
*Ad*. *furfurea*	1	1	1	*	*	*
*Ae*. *aegypti*	1	0.03	0.33	*	*	*
*Ae*. *aurovenatus*	10	0.17	0.06	5	1.35	0.44
*Ae*. *circumluteolus*	22	0.22	0.07	71	5.92	0.18
*Ae*. *cumminsii*	13	0.16	0.02	2	0.03	0
*Ae*. *durbanensis*	16	2.12	0.12	*	*	*
*Ae*. *leesoni*	8	0.14	0.15	*	*	*
*Ae*. *mcintoshi*	31	1.28	0.09	17	1.69	0.13
*Ae*. *ochraceus*	6	0.17	0.36	*	*	*
*Ae*. *sudanensis*	1	0.03	0.33	*	*	*
*An*. *funestus* group	1	0.03	0.32	*	*	*
*An*. *gambiae* complex	2	0.01	0	*	*	*
*An*. *pharoensis*	1	0.03	0.32	*	*	*
*Cx*. *antennatus*	40	0.63	0.02	*	*	*
*Cx*. *decens*/*trifoliatus*	7	0.06	0.04	*	*	*
*Cx*. *neavei*	28	4.65	0.26	*	*	*
*Cx*. *perfuscus*	1	0.48	0.42	*	*	*
*Cx*. *pipiens* complex	26	1.23	0.07	*	*	*
*Cx*. *poicilipes*	21	3.03	0.44	*	*	*
*Cx*. *simpsoni*	15	0.34	0.13	*	*	*
*Cx*. *tritaeniorhynchus*	14	0.19	0.11	*	*	*
*Cx*. *zombaensis*	1	0.03	0.32	*	*	*
*Ma*. *africana*	13	0.39	0.07	*	*	*
*Ma*. *uniformis*	35	1.99	0.06	*	*	*

The number of blood fed mosquitoes (*n*) is indicated for each habitat

*Species not collected in the location or blood meal not identified

### Molecular identification of mosquitoes

Of 30 mosquitoes requiring molecular identification, 28 species belonging to four genera and five subgenera were identified successfully using the insect *COI* gene conventional PCR. The *Aedes* genus mosquitoes consisted of mosquitoes from the subgenera *Neomelaniconion* (*Ae*. *mcintoshi*; *n* = 7) and *Aedimorphus* (*Ae*. *durbanensis*; *n* = 3 and *Ae*. (*Aedimorphus*) *leesoni*; *n* = 1). The other mosquitoes were from genus *Culex*, subgenus *Culex* (*Cx*. *antennatus*; *n* = 12 and *Cx*. (*Culex*) *telesilla*; *n* = 1), genus *Mansonia*, subgenus *Mansonioides* (*Ma*. *africana*; *n* = 2 and *Ma*. *uniformis*; *n* = 1) and genus *Aedeomyia*, subgenus *Lepiothauma* (*Ad*. *furfurea*; *n* = 1). The phylogenetic tree (Additional file 2: Fig. S2) was generated using a total of 64 sequences including 28 mosquito sequences from the study, 36 of their homologous sequences from GenBank and one sand fly (*Lutzomyia longipalpis*) as an outgroup. A phylogenetic tree was inferred by the maximum likelihood method and Tamura-Nei model [47] and the tree with the highest log likelihood (−6450.16) is shown (Additional file 2: Fig. S2). The mosquito sequences were grouped into four main clades consisting of mosquitoes from *Aedes*, *Culex*, *Mansonia*, and *Aedeomyia* genera. The largest clade contained sub-clades of all *Aedes* mosquitoes. Other mosquitoes from the *Culex*, *Mansonia*, and *Aedeomyia* genera clustered with mosquitoes from the same genus. The results from the phylogenetic analysis of the mosquito sequences further confirmed the molecular identification of the damaged mosquitoes. Genbank accession numbers for all mosquito sequences generated in this study (Additional file 3: Table S1) and other mosquitoes included are shown on the tree.

## Discussion

This study identified the vertebrate blood meal sources of blood-fed mosquitoes collected from north-eastern KZN. Floodwater mosquitoes *Ae*. *circumluteolus* and *Ae*. *mcintoshi* were the most frequently captured mosquitoes at the wildlife reserve and the rural sites, respectively. Previous studies have reported *Ae*. *circumluteolus* and *Ae*. *mcintoshi* as significant RVFV vectors in the study area and the inland plateau, respectively [5, 16]. Other collected blood-fed *Aedes* mosquitoes that are potential RVFV vectors in the study area were *Ae*. *durbanensis* and *Ae*. *ochraceus*. An unfed *Ae*. *durbanensis* tested positive for RVFV in a study that was conducted in the same area, suggesting that this mosquito species could serve as a vector for circulation of the virus if it present in abundance [48]. Rift Valley fever virus has been isolated in West and Central Africa from *Ae*. *ochraceous* and *Ae*. *cumminsii* [6], implying that these mosquitoes are potential vectors of RVFV.

*Culex antennatus* and *Cx*. *neavei*, which are both considered as potential secondary vectors in the study area [49] were the most frequently collected blood-fed members of the *Cx*. genus. Isolations of RVFV from *Cx*. *antennatus* were reported from West and Central Africa [6]. An earlier study conducted in north-eastern KZN during an RVFV outbreak in 1981, reported isolations of RVFV from *Cx*. *neavei* (*n* = 1) and *Cx*. *zombaensis* (*n* = 7) [49]. During that study, *Cx*. *zombaensis* was the most common species and the only species that yielded multiple isolations of RVFV, suggesting that it was the main vector during the outbreak [49]. Rift Valley fever virus was also isolated from *Cx*. *poicilipes* and a *Cx*. *pipiens* complex member from south-eastern Mauritania [50] and Egypt [51], respectively. The virus was also detected in *Ma*. *africana* and *Ma*. *uniformis*, which were collected during the 2006/2007 epidemic in Kenya [52].

Vector competence is an important criterion when evaluating whether a mosquito species is likely to be a vector in a particular area. It is the ability of a vector to transmit an infectious agent or the probability that an infected mosquito will cause a new infection [53]. It indicates the capacity of a vector to be infected, maintain and transmit an infectious agent [54]. Mosquito species susceptible to the virus, with the ability to successfully transmit it, and which are sufficiently abundant in an area where the virus is present may be considered as potential vectors in that area. Mosquito vectors that have been implicated in RVFV transmission in other areas and are present in abundance in an area may also be considered significant. Despite the repeated isolation of the virus from many mosquito species, their role in RVFV transmission to livestock and humans is poorly understood [15]. Determining the bloodmeal sources these vectors feed on is crucial in understanding how the virus is amplified and transmitted [28].

Our vertebrate blood meal source identification success rate of 73% is comparable to rates that have been observed in several blood meal analysis studies [55–59]. Approximately 23% of the blood meals could not be identified with the primers used. Most of these DNA samples could not be amplified, although 4.6% were amplified but failed to yield a positive identification owing to low similarity percentage to sequences from GenBank. Sequence search results are only reliable if the query sequence is closely related to the sequences available in GenBank. A high percentage cut-off value is critical to accommodate intraspecific mitochondrial DNA sequence variations among organisms [28]. Our cutoff value of 96% is comparable to those used in related studies [15, 28].

Our findings show that more blood meals were identified as mammalian (89%) than avian (11%). It has been reported that the body mass of the vertebrate blood meal source influences the host preference of some mosquitoes [13]. Larger hosts emit a larger quantity of metabolic carbon dioxide and are able to attract a wide range of mosquitoes [13]. The abundance of vertebrate blood meal sources also determines the host choice of a mosquito, particularly for opportunistic mosquitoes [60]. In addition to extrinsic factors, other intrinsic determinants, such as genetics and physiology, influence the host preference of mosquitoes [61]. The most frequent hosts identified in the present study were cattle (rural sites) and nyala (reserve), which are both common in their respective areas. In the rural study sites, *Ae*. *circumluteolus* and *Ae*. *mcintoshi* mostly fed on cattle, consistent with what has been previously reported. These important floodwater RVFV vectors were found to select cattle hosts over humans, giraffes, and other hosts in a study conducted in Kenya [14].

*Culex* mosquitoes fed on birds more often and fed on a wider range of hosts than *Aedes*. Only three *Aedes* mosquitoes fed on birds, *Ae*. *circumluteolus* (*n* = 2) on chickens, and *Ae*. *mcintoshi* (*n* = 1) on a White-browed Scrub Robin. *Culex neavei* (*n* = 14) and *Cx*. *poicilipes* (*n* = 13) fed on avian hosts in almost equal proportions. This finding was not surprising as species from the genus *Culex* have been reported to be opportunists, feeding on a wider range of hosts [62]. Other *Culex* mosquitoes, such as *Cx*. *pipiens* and *Cx*. (*Culex*) *quinquefasciatus* have been reported to be ornithophilic, feeding primarily on birds [63, 64]. All the *Ae*. *durbanensis* blood meals that were successfully identified were from the household (rural). Although *Ae*. *durbanensis* fed predominantly on cattle, two had fed on buffalo and nyala, which are found in the reserve, located approximately 6 km from the household. This finding suggests that the blood-fed mosquitoes could have dispersed from the reserve to the household. Although the dispersal distances of *Ae*. *durbanensis* have not been reported, a study that evaluated the dispersal of mosquitoes reported *Ae*. *vexans* and *Ae*. (*Stegomyia*) *albopictus* to possess strong and weak flight capacity, respectively [65]. This finding suggests that some *Aedes* mosquitoes may be capable of dispersing for longer distances. Similarly, we identified a *Cx*. *neavei* collected in the rural area (Namaneni) that had fed on a black rhinoceros, the closest of which would have been in Tembe Elephant Park (TEP), 12 km away. This suggests that this species had dispersed at least that distance, or that the rhinoceroses had strayed into the study area.

Network analysis highlights the pivotal roles played by known vectors and their hosts in the RVFV transmission cycle [22]. The reserve network had fewer nodes, and there were also fewer node interactions between the levels. Our results are comparable to what was reported in [21], although they compared the rural to the urban setting, their rural network was made up of higher numbers of both nodes and interactions between the nodes. The rural area consists of various types of temporary and permanent water bodies and artificial water collections, allowing the survival of different mosquito species [22]. Most households in the study area own various livestock and domestic species. In contrast, the reserve had a smaller variety of mosquito breeding habitats and, although waterbird nesting colonies were present in the reserve, mosquito trapping was not done near to them. Much of the reserve consisted of thick bush and forest, with a large variety of tree hole breeding mosquito species; however, these were not sampled during this study as collections were done at ground level. Thus, while our targeted approach yielded many potential RVFV vectors, it limited our ability to discuss the full mosquito community.

The connectance value of the reserve network was 0.57, suggesting that 57% of all potential host-mosquito interactions had been observed. The connectance of the rural network was 0.18, indicating that only 18% of the possible interactions between the hosts and mosquitoes were observed. Agricultural development and deforestation are common in rural areas and they have been reported to disturb ecological communities, leading to general loss of biodiversity and an increase in other groups of species [66, 67]. The strongest mosquito species in the networks have the highest diversity of hosts and included *Cx*. *neavei* and *Cx*. *poicilipes* in the rural network and *Ae*. *circumluteolus* in the reserve network. The low *d*' values (except for *Ad*. *furfurea*) obtained in this study indicate absence of evidence for vertebrate blood meal source specialization by the mosquitoes. The *d*' index is recommended over other specialization indices, such as paired difference index, as it can also estimate specialization values for singletons [22, 68]. The most common potential RVFV mosquito vectors and their hosts may serve an important role in the transmission and maintenance of RVFV in the area and may assist in spreading the virus to other areas.

The role of mammals in the maintenance of RVFV remains poorly understood. A study conducted in the study area reported the presence of RVFV antibodies and active seroconversion in goats and cattle, indicating that the virus is circulating in these species in the area [10]. A recent study found the RVFV seroprevalence in nyala (34%) and impala (46%) in NGR and TEP [69] to be comparable to that in goats (32%) and cattle (34%) in adjacent rural areas [10]. It has been proposed that wildlife may play a role in the maintenance of RVFV during interepidemic periods [70].

While we did not detect RVFV in the mosquitoes tested (*n* = 561), a recent study in the area detected RVFV in a pool of *Ae*. *durbanensis* (*n* = 4077 mosquitoes/105 pools) [48]. Our sample size of *Ae*. *durbanensis* (*n* = 16) made detecting RVFV at the rates reported in literature highly unlikely. Low RVFV detection rates in mosquitoes have been reported for *Cx*. *tritaeniorhynchus* (6 isolations from 15,428) and *Aedes vexans arabiensis* Patton (7 isolations from 8091) [71]. Other previous studies did not detect RVFV in mosquitoes [72–74], despite known virus circulation. Future blood analysis studies and RVFV screening of the potential vectors during outbreaks would provide better insights of the disease transmission dynamics. An earlier study conducted in north-eastern KZN [49] and studies from Kenya [15, 52] detected RVFV from mosquitoes, which were collected during outbreaks. To better characterize the vectors of RVFV in the study area, vector competency studies should also be conducted.

## Conclusions

This study has shown that several potential RVFV mosquito vectors are available in the area, and that they feed on multiple hosts, with some feeding on both livestock and wildlife, which may increase RVFV amplification. Most of the blood meals for floodwater *Ae*. *circumluteolus* and *Ae*. *mcintoshi* in the rural areas originated from cattle while nyala was the most common vertebrate blood meal source in the reserve and a source of blood for majority of *Ae*. *circumluteolus* meals. These results may contribute to the knowledge of the potential vectors and hosts that comprise the RVFV reservoir system. Network analysis of the host-biting communities provides insight into predicting the mosquito and blood host species that play a central role in the transmission of a vector-borne disease. The feeding patterns found for known vectors of RVFV support its inclusion in differential diagnoses for people and animals in the region.

### Supplementary Information

Additional file 1: Figure S1. Host-biting networks for A) reserve network and B) rural network of mosquitoes and their vertebrate hosts from the north-eastern KZN.

Additional file 2: Figure S2. The phylogenetic tree generated using from 64 mosquito sequences of *COI* was inferred by the Maximum Likelihood method and Tamura-Nei model with 1000 bootstrap replicates.

Additional file 3: Table S1. Generated mosquito sequences, their GenBank (Submission: SUB14487250) accession numbers, highest percentage similarity to their homologous sequences and query covers.

## Data Availability

All data generated or analyzed during this study are included in this article and its additional files.

## Appendix

See Table 4.

**Table 4:** Total numbers of blood-fed mosquito species collected from the different sites in the study area

Species	Household	Mpala	Namaneni	Ndumo GR	Total
*Ae*. *aegypti*	1	0	0	0	1
*Ma*. *africana*	2	3	15	0	20
*Cx*. *antennatus*	30	4	23	0	57
*Ae*. *aurovenatus*	10	0	0	4	14
*Cq*. *chrysosoma*	0	0	1	0	1
*Ae*. *circumluteolus*	26	5	6	91	128
*Ae*. *cumminsi*	15	0	0	2	17
*Cx*. *decens*/ *Cx*. *trifoliatus*	2	0	8	0	10
*Ae*. *durbanensis*	20	2	0	0	22
*An*. *funestus*	0	1	1	0	2
*Ad*. *furfurea*	0	1	0	0	1
*An*. *gambiae*	2	0	1	0	3
*Ae*. *leesoni*	8	0	0	0	8
*Ae*. *mcintoshi*	48	0	4	2	54
*Cx*. *neavei*	1	1	43	3	48
*Ae*. *ochraceus*	6	0	0	0	6
*Cx*. *perfuscus*	0	0	3	0	3
*An*. *pharoensis*	0	0	2	0	2
*Cx*. *pipiens* complex	19	3	9	0	31
*Cx*. *poicilipes*	2	14	16	0	32
*Cx*. *simpsoni*	11	0	14	0	25
*Mi*. (*Mimomyia*) sp.	0	0	1	0	1
*Ae*. *sudanensis*	2	0	0	0	2
*Cx*. *telesilla*	1	0	0	0	1
*Cx*. *tritaeniorhynchus*	1	7	12	0	20
*Cx*. (*Culex*) sp.	0	0	1	0	1
*Ma*. *uniformis*	5	1	37	2	45
*An*. *ziemanni*	0	2	2	0	4
*Cx*. *zombaensis*	0	0	2	0	2
Total	212	44	198	104	561
