# Supplementary material for: Characterization of mosquito host-biting networks of potential Rift Valley fever virus vectors in north-eastern KwaZulu-Natal province, South Africa
Source: Parasit Vectors. 2024 Aug 13;17:341. doi: 10.1186/s13071-024-06416-0 (PMC11323694; doi:10.1186/s13071-024-06416-0)
Supplement: Supplementary file 1 — Additional file 1: Figure S1. Host-biting networks for A) reserve network and B) rural network of mosquitoes and their vertebrate hosts from the north-eastern KZN. [file 13071_2024_6416_MOESM1_ESM.docx]

**Supplementary materials**

A) Reserve


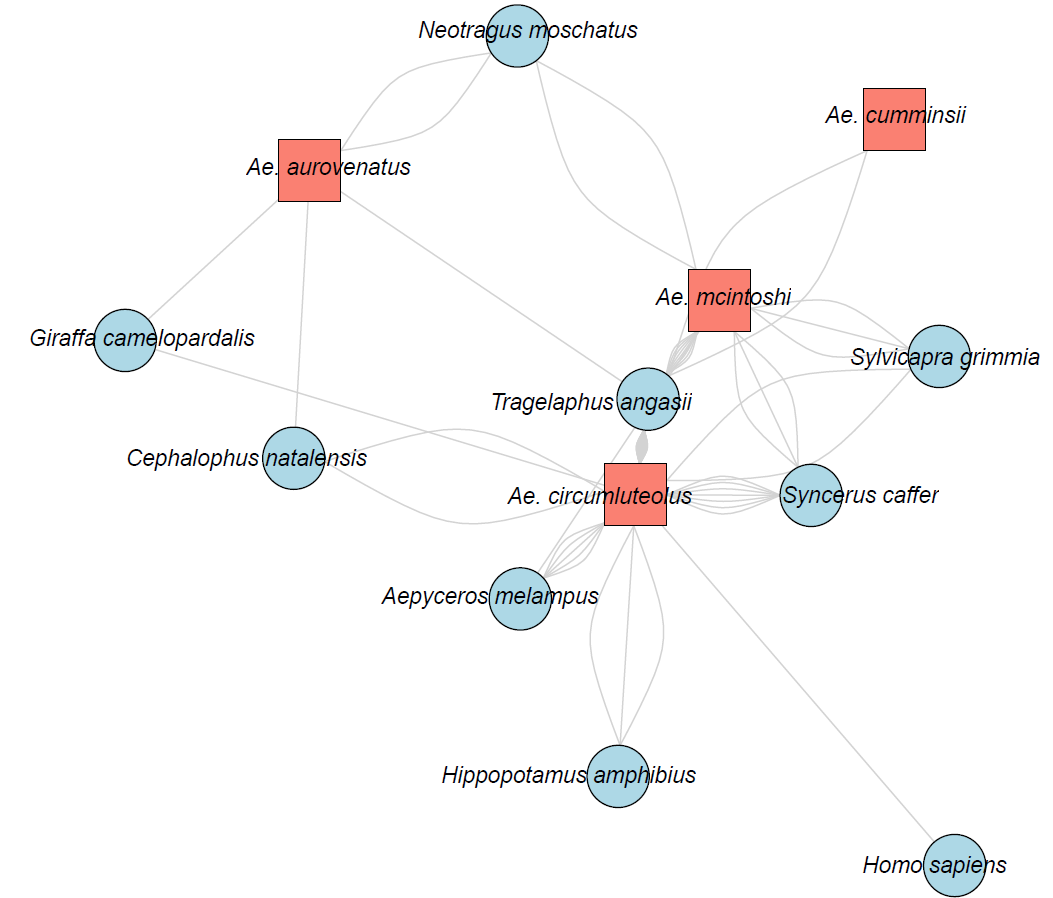


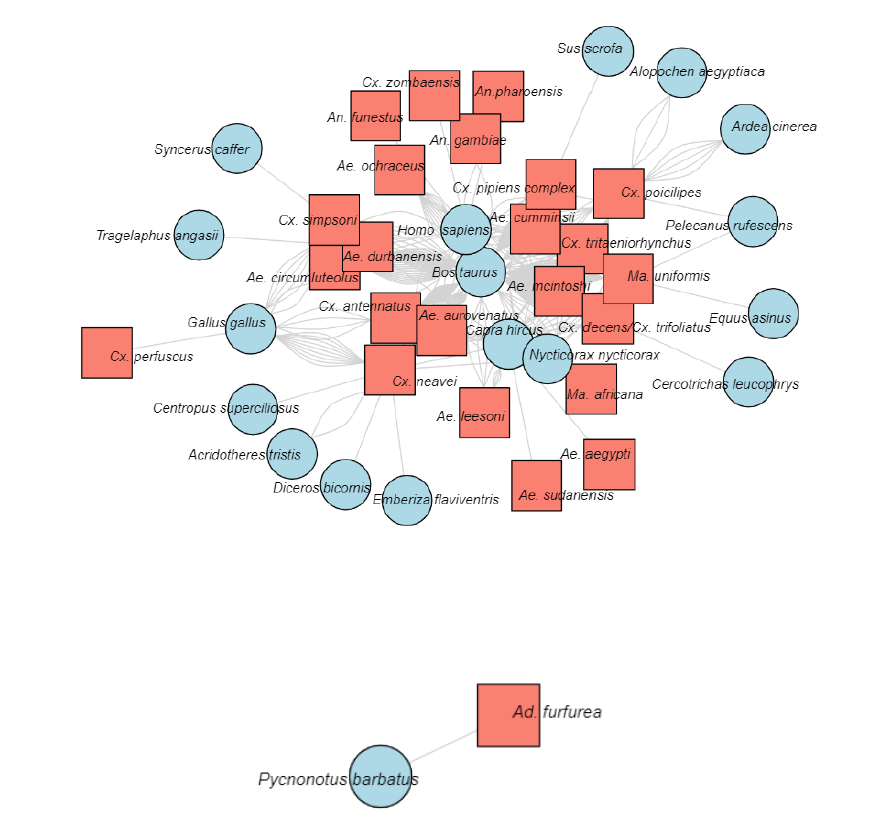


B) Rural

**Additional file 1: Figure S1.** Host-biting networks for A) reserve network and B) rural network of mosquitoes and their vertebrate hosts from the north-eastern KZN. The mosquito species are indicated on the salmon-coloured squares and the hosts on the blue circles. The mosquitoes were collected from November 2019 – February 2023.
