# Supplementary material for: Characterization of mosquito host-biting networks of potential Rift Valley fever virus vectors in north-eastern KwaZulu-Natal province, South Africa
Source: Parasit Vectors. 2024 Aug 13;17:341. doi: 10.1186/s13071-024-06416-0 (PMC11323694; doi:10.1186/s13071-024-06416-0)
Supplement: Supplementary file 2 — Additional file 2: Figure S2. The phylogenetic tree generated using from 64 mosquito sequences of COI was inferred by the Maximum Likelihood method and Tamura-Nei model with 1000 bootstrap replicates. [file 13071_2024_6416_MOESM2_ESM.docx]

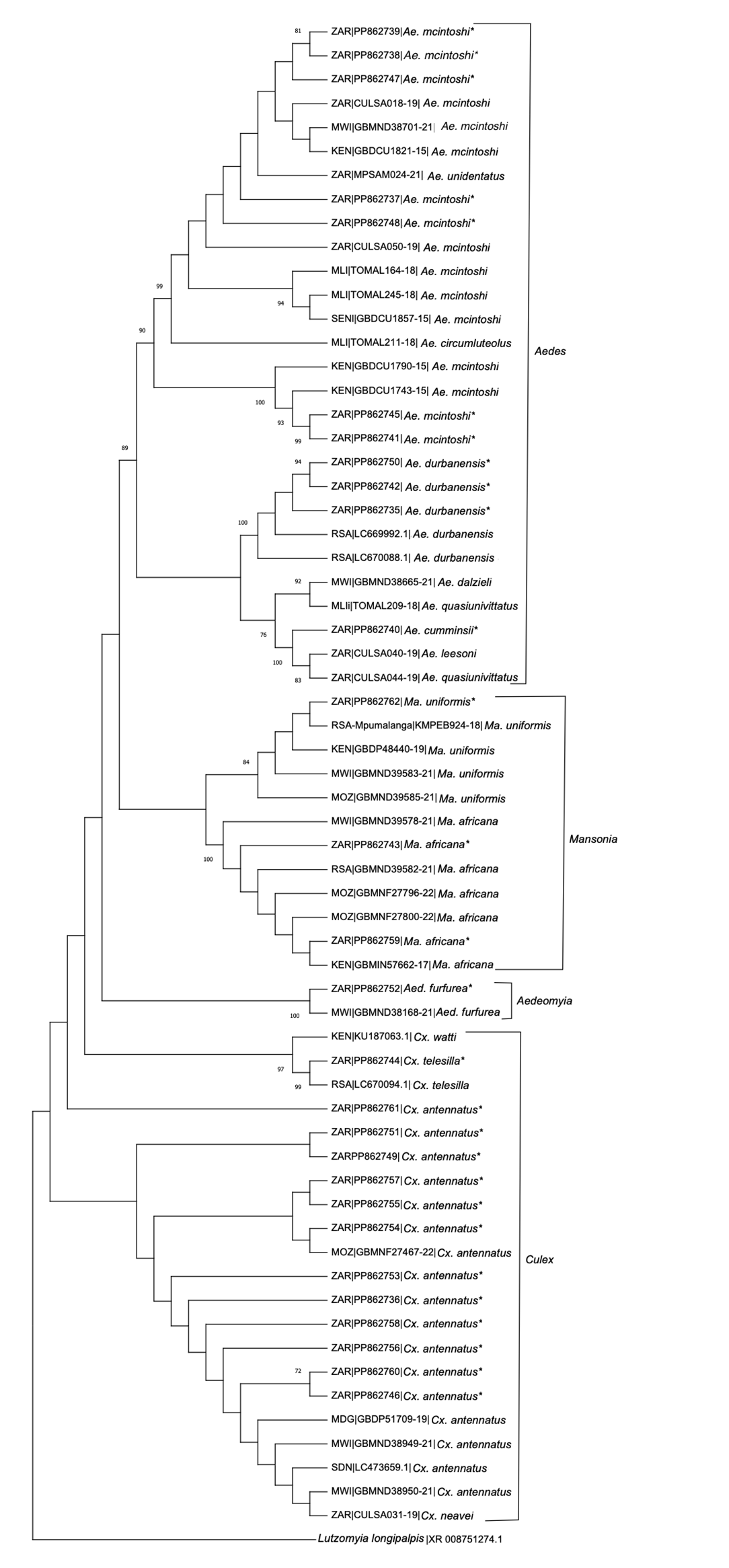


**Additional file 2: Figure S2**. The phylogenetic tree generated using from 64 mosquito sequences of CO1 was inferred by the Maximum Likelihood method and Tamura-Nei model with 1000 bootstrap replicates. Numbers next to branches indicate the branch percentage support. Samples which were part of this study are denoted by the start (*) symbol. Branch support percentages > 70% are shown. GenBank accession numbers and countries of origin are indicated for all sequences. South Africa = ZAR, Malawi = MWI, Madagascar = MDG, Mozambique = MOZ, Mali = MLI, Kenya = KEN, Senegal = SEN, Sudan = SDN
