# Supplementary material for: Characterization of mosquito host-biting networks of potential Rift Valley fever virus vectors in north-eastern KwaZulu-Natal province, South Africa
Source: Parasit Vectors. 2024 Aug 13;17:341. doi: 10.1186/s13071-024-06416-0 (PMC11323694; doi:10.1186/s13071-024-06416-0)
Supplement: Supplementary file 3 — Additional file 3: Table S1. Generated mosquito sequences, their GenBank (Submission: SUB14487250) accession numbers, highest percentage similarity to their homologous sequences and query covers. [file 13071_2024_6416_MOESM3_ESM.docx]

**Additional file 3: Table S1.** Generated mosquito sequences, their GenBank (Submission: SUB14487250) accession numbers, highest percentage similarity to their homologous sequences and query covers.

| **Mosquito species** | **GenBank accession number** | **Query coverage (%)** | **Highest identity (%)** |
| --- | --- | --- | --- |
| *Aedes durbanensis* | PP862735 | 99 | 99.8 |
| *Culex antennatus* | PP862736 | 96 | 99.5 |
| *Aedes mcintoshi* | PP862737 | 97 | 99.7 |
| *Aedes mcintoshi* | PP862738 | 99 | 99.5 |
| *Aedes mcintoshi* | PP862739 | 99 | 99.5 |
| *Aedes cumminsi* | PP862740 | 99 | 93.6 |
| *Aedes mcintoshi* | PP862741 | 99 | 95.6 |
| *Aedes durbanensis* | PP862742 | 95 | 99.7 |
| *Mansonia africana* | PP862743 | 97 | 99.1 |
| *Culex telesilla* | PP862744 | 95 | 100 |
| *Aedes mcintoshi* | PP862745 | 100 | 98.9 |
| *Culex antennatus* | PP862746 | 95 | 99.7 |
| *Aedes mcintoshi* | PP862747 | 99 | 99.7 |
| *Aedes mcintoshi* | PP862748 | 99 | 99.2 |
| *Culex antennatus* | PP862749 | 95 | 99.4 |
| *Aedes durbanensis* | PP862750 | 95 | 99.7 |
| *Culex antennatus* | PP862751 | 97 | 99.8 |
| *Aedeomyia furfurea* | PP862752 | 95 | 99.5 |
| *Culex antennatus* | PP862753 | 96 | 100 |
| *Culex antennatus* | PP862754 | 95 | 99.8 |
| *Culex antennatus* | PP862755 | 96 | 100 |
| *Culex antennatus* | PP862756 | 97 | 100 |
| *Culex antennatus* | PP862757 | 95 | 99.5 |
| *Culex antennatus* | PP862758 | 95 | 99.8 |
| *Mansonia africana* | PP862759 | 98 | 99.85 |
| *Culex antennatus* | PP862760 | 97 | 99.8 |
| *Culex antennatus* | PP862761 | 96 | 99.5 |
| *Mansonia uniformis* | PP862762 | 97 | 100 |
